# Purinergic Signaling in the Pathophysiology and Treatment of Huntington’s Disease

**DOI:** 10.3389/fnins.2021.657338

**Published:** 2021-07-01

**Authors:** Melissa Talita Wiprich, Carla Denise Bonan

**Affiliations:** ^1^Programa de Pós-Graduação em Medicina e Ciências da Saúde, Escola de Medicina, Pontifícia Universidade Católica do Rio Grande do Sul, Porto Alegre, Brazil; ^2^Laboratório de Neuroquímica e Psicofarmacologia, Escola de Ciências da Saúde e da Vida, Pontifícia Universidade Católica do Rio Grande do Sul, Porto Alegre, Brazil; ^3^Instituto Nacional de Ciência e Tecnologia em Doenças Cerebrais, Excitotoxicidade e Neuroproteção, Porto Alegre, Brazil

**Keywords:** Huntington’s disease, motor dysfunction, A_2_A receptors, adenosine, ATP, nucleotide metabolism

## Abstract

Huntington’s disease (HD) is a devastating, progressive, and fatal neurodegenerative disorder inherited in an autosomal dominant manner. This condition is characterized by motor dysfunction (chorea in the early stage, followed by bradykinesia, dystonia, and motor incoordination in the late stage), psychiatric disturbance, and cognitive decline. The neuropathological hallmark of HD is the pronounced neuronal loss in the striatum (caudate nucleus and putamen). The striatum is related to the movement control, flexibility, motivation, and learning and the purinergic signaling has an important role in the control of these events. Purinergic signaling involves the actions of purine nucleotides and nucleosides through the activation of P2 and P1 receptors, respectively. Extracellular nucleotide and nucleoside-metabolizing enzymes control the levels of these messengers, modulating the purinergic signaling. The striatum has a high expression of adenosine A_2A_ receptors, which are involved in the neurodegeneration observed in HD. The P2X7 and P2Y2 receptors may also play a role in the pathophysiology of HD. Interestingly, nucleotide and nucleoside levels may be altered in HD animal models and humans with HD. This review presents several studies describing the relationship between purinergic signaling and HD, as well as the use of purinoceptors as pharmacological targets and biomarkers for this neurodegenerative disorder.

## Introduction

Huntington’s disease (HD) is a devastating, progressive, and fatal neurodegenerative disorder inherited in an autosomal dominant manner (Smith-Dijak et al., 2019; Blumenstock and Dudanova, 2020). It is triggered by an expansion of a cytosine-adenine-guanine (CAG) triplet repeat in exon 1 of the huntingtin (*HTT*) gene, located on chromosome 4 (The Huntington’s Disease Collaborative Research Group, 1993; Capiluppi et al., 2020). This change leads to an expanded polyglutamine (polyQ) region in the encoded HTT protein (Bailus et al., 2017; Rai et al., 2019). As a result, the expressed HTT protein is a mutant (mHTT; Cybulska et al., 2020). Individuals with up to 35 CAG repeats are usually considered healthy, while people with 36 to 39 CAG repeats may or may not develop the signs and symptoms of HD (Shoulson and Young, 2011; Capiluppi et al., 2020). More than 50 CAG repeats always cause the disease (Capiluppi et al., 2020). There is an inverse correlation between the number of CAG repeats, age at onset, and the severity of HD symptoms (Bates et al., 2015; Petersén and Weydt, 2019).

It is estimated that the mean HD prevalence is 5 in 100,000 people (Baig et al., 2016; Illarioshkin et al., 2018). HD is characterized by a neurobehavioral progressive triad with motor dysfunction, psychiatric disturbance, and cognitive decline (Stahl and Feigin, 2020). The motor dysfunction is subdivided into two stages: In the early stage, there are abnormal involuntary movements, known as chorea, while in the late stage, the voluntary movements are impaired, causing bradykinesia, dystonia, and motor incoordination. The observed neuropsychiatric symptoms include depression, apathy, irritability, anxiety, and psychosis. The cognitive impairment often precedes the motor abnormalities. The cognitive alterations include impaired attention and visuospatial functions and slow planning processing speed. The cognitive decline progresses to dementia (Stahl and Feigin, 2020), and death becomes imminent 15–20 years after disease onset (Blumenstock and Dudanova, 2020). These dysfunctions can be attributed to multiple brain regions that exhibit neurodegeneration, including the cerebral cortex, thalamus, subthalamic nucleus, globus pallidus, substantia nigra, and hypothalamus. However, the hallmark of the disease is the pronounced neuronal loss in the striatum (caudate nucleus and putamen; Rubinsztein, 2002; Ramaswamy et al., 2007; Coppen and Roos, 2017). Furthermore, HD patients may develop metabolic symptoms including weight loss and cardiac and musculoskeletal dysfunction, among others (Blum et al., 2018; Croce and Yamamoto, 2019; Dufour and McBride, 2019).

**FIGURE 1:**
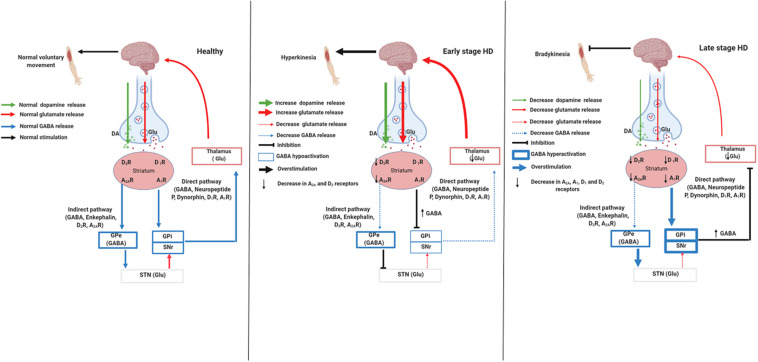
Signaling pathways involved in stages of Huntington’s disease. Created with Biorender.com.

The striatum is a region responsible for the control of many behaviors, such as movement, flexibility behavior, motivation, and learning (Koch and Raymond, 2019). Two different striatal pathways express distinct neurotransmitters and neuropeptides (Graybiel, 2000). The indirect pathway contains cholinergic interneurons that express dopamine D_2_ receptors (D_2_R), adenosine A_2*A*_ receptors (A_2A_R), and enkephalin; it projects to the globus pallidus external (Figure 1; GPe; Albin et al., 1989). This pathway acts by inhibiting voluntary movements; because the neurons are degenerated in the early stage of HD, there is a decrease in D_2_R and A_2*A*_R and thus uncontrolled voluntary movements, coinciding with chorea symptoms (Figure 1; Albin et al., 1989; Graybiel, 2000; Koch and Raymond, 2019). The direct pathway expresses spiny projection neurons (SPNs) that contain gamma-aminobutyric acid (GABA) coexisting with neuropeptide P and dynorphin. Besides, dopamine D_1_ receptors (D_1_R) project into the substantia nigra pars reticulate (SNpr) and globus pallidus internal (GPi), initiating voluntary movements (Figure 1; Albin et al., 1989). In the late stage of HD, besides the damaged indirect pathway, there is the degeneration of direct pathway neurons, a phenomenon that decreases D_1_R and cortex stimulation. This phenomenon leads to the hypokinetic symptoms, which are typical of this stage (Figure 1; Albin et al., 1989; Graybiel, 2000; Koch and Raymond, 2019).

Furthermore, neurotransmitters, such as dopamine, acetylcholine, glutamate, and GABA, are involved in motor coordination and alterations in their levels induce motor deficits. Evidence has demonstrated alterations in these neurotransmitter levels in early- and late-stage HD (Spokes, 1980; Kish et al., 1987; Jamwal et al., 2015; Jamwal and Kumar, 2019). These changes in neurotransmitter levels might cause important intracellular biochemical changes, such as a decrease in mitochondrial complex II, III, and IV activity and adenosine triphosphate (ATP) levels, calcium (Ca^2+^) overload, excitotoxicity, oxidative stress, and mitochondrial dysfunction (Johri et al., 2013; Carmo et al., 2018; Jodeiri Farshbaf and Kiani-Esfahani, 2018), triggering cell death (Liot et al., 2017). Thus, there is an imbalance in the activity between the direct and indirect pathways, resulting in an inadequate functioning of different neurotransmitter systems in HD. One of the neurotransmitter systems involved in the pathophysiology of HD is the purinergic signaling (Burnstock, 2015), mediated by the action of nucleotides and nucleosides in the P2 and P1 receptors, respectively. Both ATP and adenosine are the most important messengers in the purinergic system, which participates in the control of several behaviors (Burnstock, 2015). Adenosine acts as a neuromodulator; specifically, it modulates dopaminergic and glutamatergic neurotransmission systems (Ferré et al., 2007; Fuxe et al., 2007; Ciruela et al., 2015). Changes in ATP and adenosine levels have been observed in HD (Seong et al., 2005; Kao et al., 2017). Studies have focused on the impact of purinergic signaling on HD as well as the development of pharmacological strategies related to the purinergic system as therapies for HD (Blum et al., 2002; Chou et al., 2005; Simonin et al., 2013; Villar-Menéndez et al., 2013; Kao et al., 2017). Therefore, this review will discuss the role of purinergic signaling in HD as well as the involvement of purinoceptors in the disease progression and their relevance for application as pharmacological targets and biomarkers for HD.

## Purinergic Signaling

Adenosine triphosphate and adenosine are recognized as the most powerful purinergic signaling messengers (Burnstock, 1972). Purinergic receptors are classified into P1 and P2 according to their biochemical and pharmacological properties (Burnstock, 2018; Cheffer et al., 2018). P2 receptors are activated by purines [ATP, adenosine diphosphate (ADP)] and pyrimidines (uridine triphosphate, uridine diphosphate) and classified as P2X and P2Y receptors (Abbracchio and Burnstock, 1994; Burnstock, 2011). P2X receptors are ATP-gated ion channels permeable to sodium (Na^+^) and Ca^2+^ influx and potassium (K^+^) efflux, which leads to depolarization of the cell membrane. Seven subunits of these receptors (P2X1–7) are expressed by different cells (Burnstock, 2008). P2Y receptors are metabotropic, activated by purines and pyrimidines, and subdivided into eight receptor subtypes (P2Y_1_, P2Y_2_, P2Y4, P2Y_6_, P2Y_11_, P2Y_12_, P2Y_13_, and P2Y_14_; Burnstock, 2008; Puchałowicz et al., 2014). P1 receptors are metabotropic, selective for adenosine, and exert physiological actions through four subtypes named A_1_ (A_1_R), A_2*A*_ (A_2*A*_R), A_2*B*_ (A_2*B*_R), and A_3_ (A_3_R) (Fredholm et al., 2001; Burnstock, 2018; Ciruela, 2020). Low adenosine levels activate A_1_R and A_3_R receptors, whereas high adenosine levels activate A_2*A*_R and A_2*B*_R receptors (Borea et al., 2018). A_1_R and A_3_R activate G_*i/o*_ protein, inhibiting the production of cyclic adenosine monophosphate (cAMP), adenylate cyclase (AC), protein kinase A (PKA), and, consequently, GABA uptake. On the other hand, A_2*A*_R is activated through Gs protein that stimulates cAMP production, activating AC and PKA, increasing GABA uptake. In addition, A_2*B*_R also act through Gs protein (Sheth et al., 2014; Borea et al., 2018).

Nucleotide levels are regulated by ectonucleotidases, a group of enzymes constituted by nucleotide pyrophosphatases/phosphodiesterases (NPPs), nucleoside triphosphate diphosphohydrolases (NTPDases; CD39), alkaline phosphatase, and ecto-5′-nucleotidase (5′-NT, CD73; Bonan, 2012; Zimmermann, 2021). Ectonucleotidases promote the extracellular hydrolysis of ATP, producing ADP, adenosine monophosphate (AMP), and adenosine controlling their extracellular concentrations (Burnstock, 1980; Bonan, 2012; Cheffer et al., 2018; Cieslak et al., 2019).

Adenosine, through the action of adenosine deaminase (ADA), can be subsequently deaminated to inosine (Latini and Pedata, 2001). Inosine is phosphorylated by purine nucleoside phosphorylase (PNP) into hypoxanthine and then degraded to the stable end product uric acid (Yegutkin, 2008; Ribeiro et al., 2016). Adenosine levels are also regulated by unidirectional and bidirectional transporters, which allow nucleosides to move between the intracellular and extracellular compartments (Fredholm et al., 2001; Ribeiro et al., 2016; Stockwell et al., 2017). Finally, the action of nucleosides is then limited either through their conversion to other products of purine catabolism or through re-synthesis into nucleotides (Ribeiro et al., 2016).

## The Role of P1 Receptors in Huntington’s Disease

As mentioned above, adenosine mediates its effect through four adenosine receptors (A_1_, A_2*A*_, A_2*B*_, and A_3_). A_1_R and A_2*A*_R are mainly involved in the central effects of adenosine (Ciruela et al., 2015). A_1_R is the most abundant receptor in the brain–widely expressed in the hippocampus, cerebellum, thalamus, brain stem, and spinal cord–whereas A_2*A*_R is concentrated abundantly in the striatum (Burnstock, 2008; Borea et al., 2018).

It is known that A_1_R can interact with D_1_R to form heterodimers. Pre-synaptic A_1_R activation causes depression of excitatory transmission through Ca^2+^ channel inhibition and neuronal hyperpolarization by regulation of potassium channel. This action leads to a reduction in the release of many neurotransmitters, including acetylcholine, glutamate, dopamine, noradrenaline, and serotonin; this reduction may be beneficial in some central nervous system diseases (Yoon and Rothman, 1991; Von Lubitz et al., 1994; Gundlfinger et al., 2007).

A_2*A*_ receptors can co-localize with D_2_R, resulting in A_2*A*_R/D_2_R heteromers, which have a crucial role in the modulation of motor function. Thus, A_2*A*_R activation decreases the affinity and function of D_2_R for agonist or antagonist drugs (Borea et al., 2018). In addition, A_2*A*_R plays an important role in facilitating glutamate release, potentiating their effects via *N*-methyl-D-aspartate (NMDA) receptors as well as other neurotransmitters, such as GABA, glycine, acetylcholine, noradrenaline, and serotonin (Cunha, 2005; León-Navarro et al., 2018). In addition, there is functional cooperation between A_2*A*_R and A_1_R (heteromers), leading to antagonist actions on dopamine release–that is, when A_1_R is stimulated it inhibits dopamine release, which would oppose the stimulating effects of A_2*A*_R through action on striatal D_2_R (León-Navarro et al., 2018). A_2*A*_R can also form heterodimers with glutamate receptors (metabotropic 5 subtype [mGlu5]). The A_2*A*_R/mGlu5 heterodimers exert a synergistic inhibitory effect on dopamine binding to D_2_R (León-Navarro et al., 2018). Together, these findings lead to the hypothesis that dysfunction in adenosine receptors may cause an imbalance between dopaminergic, glutamatergic, and GABAergic neurotransmission systems, which would explain pathological processes underlying HD. This hypothesis is supported by several studies that focus on adenosine receptors, mainly A_2*A*_R, and to a lesser extent A_1_R. Here we described the involvement of A_1_R and A_2*A*_R in the progression of HD.

### A_1_R in Huntington’s Disease

The first study investigating the involvement of P1 receptors in HD was focused on the analysis of the A_1_R subtype in the striatum and parietal–frontal culture of post-mortem cerebral samples from HD subjects. Using a selective A_1_R agonist–[3N] (N6-cyclohexyl)-adenosine–the authors demonstrated a 60% decrease in A_1_R with an increase in the affinity of the binding drug for the receptor in the striatum (Whitehouse et al., 1985). Later, Matusch et al. (2014) quantified, through position emission tomography (PET) imaging, the cerebral binding of A_1_R in HD subjects subdivided into four groups: pre-manifest individuals far from (pre-HD-A) or pre-manifest individuals near (pre-HD-B) the predicted symptom onset, manifest HD patients, and controls. The results demonstrated a decrease in caudate and putaminal volumes from pre-HD and HD patients compared with control, more A_1_R in the thalamus of pre-HD-A individuals compared with control, and less A_1_R in caudate and amygdala in all stages of the disease. There was also a strong direct correlation between A_1_R with the years since disease onset–that is, the more advanced the disease, the larger the loss of A_1_R (Matusch et al., 2014). This finding indicates that A_1_R is involved in the pathogenesis of HD and might be a biomarker in specific brain areas for HD progression.

To shed further light on the possible role of A_1_R in HD, pharmacological and genetic mouse models have been used to understand the functionality of these receptors. In a pharmacological model of HD induced by 3-nitropropionic acid (3-NPA)–the main toxin used to induce an HD-like phenotype in an animal model through mitochondrial inhibition–the acute (two or three injections) and chronic (8 days) treatment with A_1_R agonist adenosine amine congener (ADAC) provoked different behavioral and neurochemical responses in mice (Blum et al., 2002). In the study, animals that received two injections of ADAC were called ADAC_4/5_, whereas animals that received three injections were called ADAC_3/5_. ADAC_4/5_ and ADAC_3/5_ treatment attenuated dystonia of hindlimbs caused by 3-NPA, while the ADAC_4/5_ group showed increased striatal succinate dehydrogenase (SDH) activity inhibited by 3-NPA and a reduced striatal lesion volume (Blum et al., 2002). Chronic ADAC treatment did not alter the motor symptoms or striatal lesion size, and there was no increase in the SDH activity (Blum et al., 2002). Interestingly, chronic treatment dramatically decreased the A_1_R density in the striatum and cortex, whereas acute treatment did not modify the A_1_R density (Blum et al., 2002).

**FIGURE 2:**
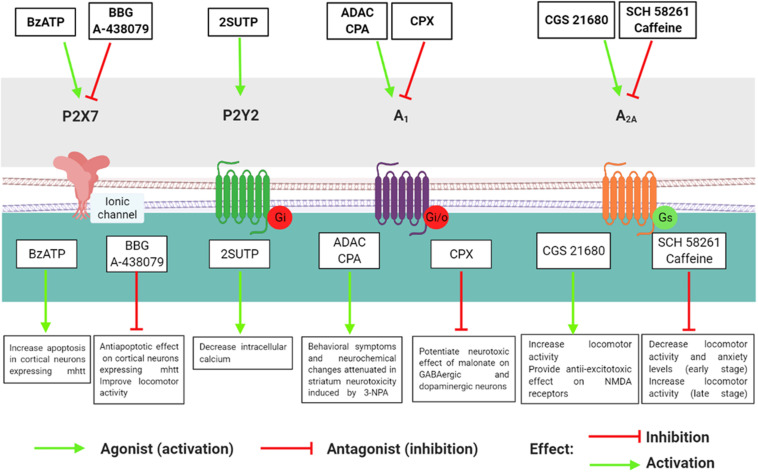
Effects of P2 and P1 agonists and antagonists in models of Huntington’s disease.

Consistently, in R6/2 mice–the most widely used transgenic model of HD–A_1_R was significantly reduced in the cortex and striatum compared with the wild type (WT) group (Ferrante et al., 2014). Moreover, corticostriatal slices of R6/2 treated with the selective A_1_R agonist cyclopentyladenosine (CPA) showed a marked reduction in synaptic transmission, with consequent inhibition of glutamate release in the pre-synaptic terminal (Ferrante et al., 2014). On the contrary, in a pharmacological model of HD induced by malonate, Alfinito et al. (2003) observed that intraperitoneal and intrastriatal A_1_R blockade with the A_1_R antagonist CPX potentiated the effect of malonate, reducing striatal dopamine levels, tyrosine hydroxylase (TH) content, and GABA levels as well as GABAergic and dopaminergic neuronal loss (Alfinito et al., 2003). Together, these findings lead to the hypothesis that the pharmacological therapy with A_1_R agonists may be a beneficial protective treatment in the early stage of HD (Figure 2). However, A_1_R antagonist drugs did not seem to be an alternative for the treatment of HD due to the crosstalk with GABAergic neurons, which enhanced the susceptibility to toxic insults, reducing A_1_R in these two populations of neurons.

### A_2*A*_R Alterations in Huntington’s Disease Progression

#### A_2*A*_R: Focus on Human Studies

Contrary to A_1_R, the role of A_2*A*_R in HD has been more widely investigated in human and animal models. The first evidence for the involvement of A_2*A*_R in HD was provided by autoradiographic mapping using the selective A_2*A*_R agonist ligand CGS 21680 in brain sections from HD patients and controls without the pathology. The results showed a dramatic decrease in A_2*A*_R in the caudate nucleus, putamen, nucleus accumbens, olfactory tubercle, and globus pallidus lateral in HD compared with control samples (Martinez-Mir et al., 1991). In a subsequent study, analyses of post-mortem neuronal tissue in HD subjects in the early, intermediate, and late stages of the disease showed A_2*A*_R bound the selective A_2*A*_R agonist ligand CGS 21680 within the caudate nucleus and putamen of the control brain. In the early and intermediate stages of HD, there was a dramatic loss of A_2*A*_R binding, whereas in brain tissue with the late stage of the disease there was no detectable A_2*A*_R binding (Glass et al., 2000).

On the other hand, higher A_2*A*_R levels are detected in peripheral blood cells, such as platelets, lymphocytes, and neutrophils at pre-symptomatic, early, and late stages of HD patients (Varani et al., 2003; Maglione et al., 2005). In addition, the higher A_2*A*_R levels in platelets correlated with CAG expansion and with anticipation in years since the onset of symptoms (Maglione et al., 2005, 2006). Thus, these findings indicate that there are A_2*A*_R alterations in the peripheral and central nervous systems in HD patients, and A_2*A*_R in blood cells might be an easy biomarker to detect and monitor HD progression.

Genetic studies support the relationship between A_2*A*_R and HD pathology. Two similar cohort studies performed in France and Germany with HD subjects showed gene polymorphisms in the A_2_*_*A*_R* gene (*ADORA2A*), mainly the rs5751876 variant, which was associated with a variation in the age at onset on the disease (Dhaenens et al., 2009; Taherzadeh-Fard et al., 2010). Compelling evidence showed increased methylcytosine and decreased 5-methylhydroxylation (enzymes responsible by DNA methylation) in the 5′ untranslated region (UTR) of *ADORA2A* in the putamen of HD patients compared with their respective controls (Villar-Menéndez et al., 2013). These data suggest that a dysfunction in A_2*A*_R gene expression might also contribute to the pathophysiology of HD.

Some habitually consumed drinks and foods–such as coffee, tea, chocolate, and soda–contain caffeine, a non-selective A_1_R and A_2*A*_R antagonist (Chen and Chern, 2011). Supported by the evidence that adenosine receptor antagonists could exert beneficial effects in neurodegenerative diseases, Simonin et al. (2013) raised the hypothesis that caffeine may be a life-style modifier in HD. For this reason, a retrospective study evaluated a possible relationship between caffeine consumption and age at onset in 80 subjects with HD. The data showed >190 mg/day caffeine consumption was significantly associated with an earlier age at onset of the disease (Simonin et al., 2013). This finding leads to the hypothesis that this association may not be a relationship with food habits but might be related to genetic determinants, such as *ADORA2A* or CYP1A genes, that may cause an earlier age at onset of the disease, influence a higher caffeine intake, and modulate behavioral effects.

#### A_2*A*_R: Evidence in Animal Models

Changes in A_2*A*_R gene expression and density have been found in transgenic animal models of HD. R6/2, HD100, and tgHD mice at 4 weeks to 24 months of age had a decrease in A_2*A*_R levels and density (Cha et al., 1999; Chan et al., 2002; Bauer et al., 2005; Tarditi et al., 2006); R6/2 animals less than 3 weeks of age had an increase in A_2*A*_R levels and density (Tarditi et al., 2006). In contrast, Benn et al. (2007) did not find alterations in A_2*A*_R gene expression in the YAC128 genetic model of HD (Benn et al., 2007). These results suggest that alterations in gene expression A_2*A*_R could have an association with HTT length and age of disease.

Several studies with controversial results have evaluated whether A_2*A*_R genetic and pharmacological activation or blockade affects behavioral symptoms and/or striatal degeneration in genetic or pharmacological models of HD. A_2*A*_R knockout between 12 and 21 weeks of age in the N171-82-Q transgenic model of HD had a deleterious impact on survival, motor coordination, body weight, striatal volume, and enkephalin and neuropeptide messenger RNA (mRNA) levels (Mievis et al., 2011). On the other hand, A_2*A*_R knockout in two R6/2 transgenic lines of HD (CAG120 and CAG240) did not cause working memory deficits and locomotor impairment compared with HD CAG120 and HD CAG240 WT mice (Li et al., 2015). Moreover, pharmacological blockade of A_2*A*_R with 1 mg/kg of the antagonist KW60002 in R6/2 (CAG240) mice at 3 months of age also reversed the working memory deficits compared with R6/2 (CAG240) mice that did not receive KW60002 (Li et al., 2015). Consistent with that finding, Fink et al. (2004) reported that A_2*A*_R genetic inactivation is beneficial to the HD animal model. The authors used two HD mouse models on different genetic backgrounds, namely C57BL/6 and 129-Steel; these models had A_2*A*_R knockout (KO; C57BL/6-A_2*A*_-KO and 129-Steel-A_2*A*_-KO). C57BL/6-A_2*A*_-KO mice and their respective WT mice at 6 months of age were administered 3-NPA twice daily for 5 days. C57BL/6-WT mice developed bradykinesia with dystonic movements and bilateral striatal lesion compared with C57BL/6-A_2*A*_-KO mice (Fink et al., 2004). Furthermore, in the 129-Steel line, 3-NPA administration also resulted in bilateral striatal lesions in WT compared with A_2*A*_R KO (Fink et al., 2004).

More recently, Domenici et al. (2018) investigated *in vivo* and *in vitro* the effect of 3-NPA in a transgenic rat strain overexpressing A_2*A*_R. Surprisingly, there was a reduction in striatal lesion volume induced by 3-NPA in transgenic rats overexpressing A_2*A*_R compared with WT animals. There was greater striatal cell viability in transgenic rats overexpressing A_2*A*_R than WT rats after exposing corticostriatal slices to 10 mM 3-NPA. In addition, 3-NPA treatment depressed synaptic transmission in corticostriatal slices of WT animals compared with rats overexpressing A_2*A*_R (Domenici et al., 2018). These data lead to the idea that A_2*A*_R is an important target involved in the pathophysiology of HD and A_2*A*_R modulation might be beneficial to attenuate the neurodegenerative progression of the disease.

A_2*A*_ receptors modulation has demonstrated beneficial effects against neurodegeneration and HD-like behavioral symptoms induced by quinolinic acid and 3-NPA. The behavioral symptoms like hyperkinesia (increased locomotor activity) and the increase in anxiety levels persistent in early stage HD were decreased by blocking A_2*A*_R using two A_2*A*_R antagonists (SCH 58261 and caffeine; Popoli et al., 2002; Scattoni et al., 2007; Mishra and Kumar, 2014). In addition, the bradykinesia (decrease in locomotor activity) present in late stage of the disease was reverted with the use of SCH 58261 and caffeine (Mishra and Kumar, 2014; Bortolatto et al., 2017). Blocking A_2*A*_R using SCH 58261 and caffeine protected against the elevated glutamate extracellular levels, excessive stimulation of NMDA receptors, increased reactive oxygen species, oxidative stress, and mitochondrial dysfunction (Popoli et al., 2002; Mishra and Kumar, 2014; Bortolatto et al., 2017). Furthermore, Fink et al. (2004) showed that striatal neurotoxicity induced by 3-NPA in C57BL/6 mice resulted in bilateral striatal lesions, while mice pre-treated with the A_2*A*_R antagonist caffeine before 3-NPA injections did not show striatal lesions.

A_2*A*_ receptors pharmacological blockade also attenuated the levels of cyclooxygenase (COX-2), prostaglandin E2, and brain-derived neurotrophic factor, a member of the neurotrophin family that participates in synaptic transmission and regulates neuronal proliferation and survival (Minghetti et al., 2007; Potenza et al., 2007). In R6/2 mice, A_2*A*_R blockade prevented alterations in anxious responses and abolished the increase in NMDA-receptor-induced striatal toxicity (Domenici et al., 2007). However, A_2*A*_R antagonism had a potentially detrimental effect on neurotoxicity induced by quinolinic acid in an animal model of HD; specifically, there was increased striatal glutamate outflow (Gianfriddo et al., 2003).

Considering that a pronounced loss of A_2*A*_R occurs in HD, researchers have tested whether A_2__*A*_ agonists could ameliorate HD symptoms. The selective A_2*A*_R agonist CGS 21680 reverted the hypolocomotor profile and the increase in the ventricle/brain ratio in R6/2 mice (Chou et al., 2005). CGS 21680 also attenuated the NMDA toxic effects in corticostriatal slices (Martire et al., 2007) and reduced the expression of NMDA receptor subunits NR1 in the striatum of R6/2 and WT mice. In addition, there was a decrease in the NR2A/NR2B ratio in the striatum of WT mice, inducing a pro-excitotoxic effect, whereas the NR2A/NR2B ratio was increased in HD mice (R6/2), and thus the NMDA receptors provided an anti-excitotoxic effect (Ferrante et al., 2010). Finally, in the liver of R6/2 mice, CGS 21680 treatment also had a protective effect, suppressing mHTT aggregates and decreasing the levels of crucial transcription factor and protein chaperones (Hsp27 and Hsp70; Chiang et al., 2009). Taken together, it is possible to suggest that A_2*A*_R alterations in HD occur in the central nervous system as well as other tissues. Thus, pharmacological modulation of A_2*A*_R through agonist or antagonist administration might exert a neuroprotective effect against HD progression (Figure 2), considering the stage of the disease, drug dosage, and period of pharmacological administration.

## P2 Receptor Involvement in Huntington’s Disease

Contrary to P1 receptors, there are few studies about P2 receptors in HD and their roles in this neurodegenerative disease remain elusive. Díaz-Hernández et al. (2009) investigated P2X7 receptor involvement in HD using two genetic mouse models of HD (Tet/HD94 and R6/1). First, cortical and striatal neurons showed an increase in P2X7 receptor protein and mRNA levels in Tet/HD94 and R6/1 compared with WT mice (Díaz-Hernández et al., 2009). Moreover, transgenic HD mice had higher P2X7 receptor levels in synaptosomes and were more sensitive to the P2X7 agonist BzATP than WT mice. This increase in sensitivity induced by BzATP led to apoptosis of cultured cortical neurons expressing mHTT, which was prevented by the P2X7 antagonist brilliant blue (BBG) (Díaz-Hernández et al., 2009). Due to the protective effect of BBG in neuronal culture, the authors investigated the efficacy of P2X7 antagonists BBG and A-438079 in R6/1 mice and their WT on body weight and locomotor parameters. BBG administration fully prevented body weight loss and significantly improved motor coordination and locomotor performance in R6/1 compared with WT mice. The antagonist A-438079 also prevented body weight loss and improved locomotor parameters in R6/1 mice, but the effect was only moderate (Díaz-Hernández et al., 2009). These data support the hypothesis that changes in P2X7 receptor levels and function lead to an increase in Ca^2+^ permeability; this eventuality could induce excitotoxicity and oxidative stress in neurons, changes that are related to HD pathogenesis (Figure 2). In the future, studies using P2X7 receptor antagonists should be performed because these compounds may have therapeutic potential for HD treatment.

Martire et al. (2021) investigated the expression and the functioning of P2X7R in two genetic models of HD: ST14A rat striatal cells, expressing full-length wild-type (WT, Q15) or mutant (Q120) htt and R6/2 mice, which resembles to juvenile forms of HD. In the presence of HD mutation, there is an altered P2X7R expression and a larger P2X7R response to the agonist BzATP, inducing cell death and reducing synaptic transmission. BzATP effect observed in the electrophysiology experimental setting are dependent of A_1_R activation. These findings may permit a better understanding of the P2X7R mechanisms and evaluate the therapeutic potential of P2X7 antagonists in HD. Certainly, this topic requires a deepen investigation since recent studies demonstrated changes in P2X7R in the brain of HD subjects (Ollà et al., 2020). Ollà et al. (2020) observed that the protein levels of the full-length form of P2X7R, also called P2X7R-A, as well as the exclusively human naturally occurring variant lacking the C-terminus region, named P2X7R-B, are upregulated. These augmented protein levels can be explained by elevated P2X7R mRNA levels. In addition, P2X7R introns 10 and 11 are more retained in HD subjects when compared with controls patients (Ollà et al., 2020). Therefore, further studies are required to evaluate the implications of P2X7R in HD and if this receptor may be a target for the development of new pharmacological therapies for this pathology.

Regarding to other P2 receptors, Glaser et al. (2020) analyzed whether P2Y_2_ receptors might be involved in the pathogenesis of HD. They investigated the role of the P2Y_2_ receptor and spontaneous Ca^2+^ concentrations in embryonic stem cells in *in vitro* HD models and controls. In basal state HD, there were higher intracellular Ca^2+^ levels than controls. Besides, there was elevated P2X7 and P2Y_2_ receptor levels in the HD cell line compared with the controls (Glaser et al., 2020). Moreover, the cells from controls (WT cells) were responsive to the P2X7 receptor agonist BzATP and the P2Y_2_ receptor agonist 2SUTP, while HD cells (expressing mHTT) showed a decrease in the 2SUTP response, with impaired P2Y_2_ receptor activation (Glaser et al., 2020). The administration of ATP or 2SUTP in HD cells decreased the concentration of intracellular Ca^2+^ transients (Glaser et al., 2020). Therefore, these results indicate that P2 receptors contribute to the pathogenesis of HD (Figure 2) and provided the first evidence for the involvement of the P2Y_2_ receptor in this neurodegenerative disease.

Together, the above-mentioned studies shed new light on the mechanism underlying HD, leading to the idea that pharmacological therapies based on P2X7 and P2Y_2_ receptors should be better investigated for HD treatment.

## Focusing on the Relationship Between Nucleotide and Nucleoside Metabolism and Huntington’s Disease

Reduced mitochondrial ATP levels and ATP/ADP ratios have been found in striatal cells containing mHTT (Seong et al., 2005). It is known that ATP depletion occurs in HD, so researchers have investigated nucleotide and nucleoside metabolism in this pathological condition. Researchers have demonstrated reduced mitochondrial ATP levels in cortical cells from an animal model of HD induced by 3-NPA, in cardiac tissue from two genetic models of HD (R6/2 and HdhQ150), and in a HEK293T cell line containing mHTT (Riepe et al., 1994; Toczek et al., 2016a, 2018). There was a decrease in the ATP/ADP ratio in cardiac tissue from R6/2 and HdhQ150 mice (Toczek et al., 2016a). There was also a reduction in ATP and AMP degradation in the HEK293T cell line containing mHTT (Toczek et al., 2018). Furthermore, studies in cardiac and cerebral tissue have shown no alteration in ADP levels in R6/2 and HdhQ150 mice. However, AMP levels were increased in R6/2 mice (Toczek et al., 2016a; Kao et al., 2017).

A genetic study of HD using different lines, including Tg51, zQ175, and R6/2, and a pharmacological model of HD induced by quinolinic acid and 3-NPA demonstrated a decrease in adenosine levels in the striatum of Tg51, zQ175, R6/2 lines, and HD animals induced by quinolinic acid and 3-NPA (Gianfriddo et al., 2003; Guitart et al., 2016; Jamwal and Kumar, 2016; Kao et al., 2017). Importantly, researchers have also found a decrease in adenosine levels in the cerebrospinal fluid of patients with HD compared with controls (Kao et al., 2017). Kao et al. (2017) observed that ATP was indirectly correlated with the number of CAG repeats and had a direct correlation with the age at onset of the disease while the adenosine/ATP ratio was negatively correlated with the disease duration of patients with HD (Kao et al., 2017).

Consistently, there was a decrease in ecto-5′-nucleotidase (an enzyme that converts AMP to adenosine) activity in HEK 293T cell line containing mHTT and cardiac tissue from R6/2 mice (Toczek et al., 2016b, 2018), while there was an increase in ADA activity in cardiac tissue from R6/2 mice (Toczek et al., 2016b). Furthermore, in the striatum of rats treated with 3-NPA, there was a decrease in inosine and hypoxanthine levels compared with the control group (Jamwal and Kumar, 2016). On the other hand, Toczek et al. (2016a) found an increase in inosine, hypoxanthine, xanthine, uric acid, and uridine levels in cardiac tissue in R6/2 and HdhQ150 mice (Toczek et al., 2016a).

Genes involved in purine metabolism (*Entpd2*, *Ampd3*, *Pnp*, and *Xdh*), adenosine metabolism (Ada), conversion of adenine nucleotides (adenylate kinase 1 [*Ak1*] and inosine monophosphatase dehydrogenase 2 [*Impdh2*]), and equilibrative nucleoside transporter-like ENT1 and ENT2 were altered in cardiac tissue, striatum, and skeletal muscle of R6/2 mice of HD (Toczek et al., 2016a; Kao et al., 2017; Mielcarek et al., 2017). Moreover, in HD patients at the early stage of the disease, there was an upregulation of the gene that encodes ENT1 (Guitart et al., 2016). Finally, nitrobenzylthioinosine (NBTI), a selective inhibitor of ENT1, and dipyridamole (DPR), a non-specific inhibitor of ENT1 and ENT2, were intrastriatally perfused in R6/2 mice. Administration of NBTI alone increased adenosine levels 1-h post-treatment, whereas both NBTI and DPR administration increased adenosine levels 1-h post-treatment, sustaining the effects up to 5 h post-treatment (Kao et al., 2017).

Regarding to studies in HD patients, it has been observed differences in the metabolism of nucleoside and its derivates. In plasma samples from HD patients, there was a significant increase in hypoxanthine and uridine levels, but there were not changes in uric acid levels compared with healthy subjects (Toczek et al., 2016a). Interestingly, hypoxanthine and uridine levels were directly correlated with HD duration and indirectly correlated with motor scores and chorea intensity (Toczek et al., 2016a). In contrast, Corey-Bloom et al. (2020) recently investigated uric acid levels in plasma and saliva samples from HD subjects and normal controls, as well as in post-mortem prefrontal cortical samples from HD subjects and controls. The authors surprisingly revealed that plasma and salivary uric acid levels were significantly lower in female pre-manifest HD and manifest HD subjects compared with normal controls, whereas salivary levels of uric acid were also significantly lower in male manifest HD subjects than controls. In male HD patients, plasma and salivary uric acid levels were negatively correlated with total functional capacity, while there were direct correlations with the total motor score. Female HD patients showed a direct correlation between plasma uric acid levels and total functional capacity, while salivary uric acid levels were significantly correlated with disease burden. Finally, in post-mortem prefrontal cortical samples from HD subjects, there was a decrease in uric acid levels (Corey-Bloom et al., 2020).

Overall, these findings indicate that purinergic signaling plays an important role in HD by inhibiting the cascade of ATP hydrolysis, modulating the concentration of adenine nucleotides and nucleosides.

## Conclusion

This review sheds light on the important regulatory role of purinergic signaling in HD pathophysiology. Notably, ATP, adenosine, and A_2*A*_R are the main actors in HD. Although some results in animal models and HD patients are controversial–depending on the model tested, type of pharmacological treatment, and period of drug administration–pharmacological modulation of A_2*A*_R, through agonist and antagonist drugs, has shown a neuroprotective effect by attenuating the behavioral symptoms and improving neurochemical parameters during HD progression. Besides, A_2*A*_R in the central and peripheral nervous system might be considered a powerful biomarker for HD progression and ought to be used in clinical practice. A_2*A*_R heterodimerizes with several other G-protein coupled receptors involved in striatal dysfunction and degeneration in HD; thus, A_2*A*_R could be considered a target for the development of pharmacological therapies for HD patients.

Several small molecules acting as A_2*A*_R antagonists have already been developed and tested in patients several neurological diseases, such as Parkinson’s Disease. Although the efficacy of these agents in Parkinson’s disease was not proved, the use of antagonists targeting A_2*A*_R in cancer immunotherapy has been also investigated. The treatment with small molecules or mAbs aiming to block adenosine signaling, either by limiting its production or its binding to adenosine receptors, has yielded important tumor control in pre-clinical studies. Moreover, simultaneous blockade of adenosine production and receptor binding, achieved by an anti-CD73 mAb co-administered with an A_2*A*_R antagonist, for example, have demonstrated synergy. Therefore, it is important to deepen the investigation of A_2*A*_R as a target for the development of existing or new agents targeting this axis, along with further testing of combinatorial strategies, which may be relevant in the search for pharmacological therapies for HD patients. Moreover, the role of A_1_R and P2 receptors in HD pathogenesis needs to be reconsidered; there should be more specific investigation on these receptors, because they could provide a powerful contribution to understanding the mechanism underlying HD. In addition, nucleoside metabolism and the control of hypoxanthine, xanthine, and uric acid levels should also be deeply investigated, because these nucleosides might be important candidates for future drugs or biomarkers for HD. In summary, purinergic signaling represents a promising research area, and the main players, such as ATP, adenosine, and A_2*A*_R, as well as the respective coadjutants, such as A_1_R, P2 receptors, and other components of nucleotide and nucleoside metabolism, should be considered possible targets for drug development for HD treatment.

## Author Contributions

Both authors contributed for the conceptualization, performed the literature review, read and approved the submitted version. MW wrote the original draft of the manuscript. CB wrote and review and editing the final version of the manuscript.

## Conflict of Interest

The authors declare that the research was conducted in the absence of any commercial or financial relationships that could be construed as a potential conflict of interest.
